# RETRACTION: Suppression of KIF22 Inhibits Cell Proliferation and Xenograft Tumor Growth in Tongue Squamous Cell Carcinoma

**DOI:** 10.1155/bmri/9874693

**Published:** 2025-07-11

**Authors:** BioMed Research International

RETRACTION: Y. Liu, R-H. Li, G. Ren, and J. Jiang, “Suppression of KIF22 Inhibits Cell Proliferation and Xenograft Tumor Growth in Tongue Squamous Cell Carcinoma,” *BioMed Research International* 2020 (2020): 6387545, https://doi.org/10.1155/2020/6387545.

The above article, published online on 22 January 2020 in Wiley Online Library (http://wileyonlinelibrary.com), has been retracted by agreement between the Section Editor of the journal, Gerald Brandacher; and John Wiley & Sons Ltd.

The retraction has been agreed following an investigation of the concerns raised by *Hoya camphorifolia* on PubPeer [1], which identified an instance of figure duplication.

Specifically:

- Figure 4a: The second and third tumors in top row and the first tumor in the bottom row appear identical to the third and fourth tumors in the top row and the second tumor of the bottom row in Figure 4a of [2].

As a result of the investigation, the data and conclusions of this article are considered unreliable.

Yi Liu disagrees with the retraction. All other authors have been informed of this decision but did not provide a response.
